# Genome-wide analysis of the *WOX* gene family and the role of *EjWUSa* in regulating flowering in loquat (*Eriobotrya japonica*)

**DOI:** 10.3389/fpls.2022.1024515

**Published:** 2022-11-03

**Authors:** Yuanhui Yu, Miaomiao Yang, Xinya Liu, Yan Xia, Ruoqian Hu, Qingqing Xia, Danlong Jing, Qigao Guo

**Affiliations:** ^1^ Key Laboratory of Horticulture Science for Southern Mountains Regions of Ministry of Education, College of Horticulture and Landscape Architecture, Southwest University, Chongqing, China; ^2^ State Cultivation Base of Crop Stress Biology for Southern Mountainous Land of Southwest University, Academy of Agricultural Sciences, Southwest University, Chongqing, China

**Keywords:** loquat, *WOX* gene family, WUS transcription factor, flowering time, protein interaction

## Abstract

The WUSCHEL (WUS)-related homeobox (*WOX*) gene family plays a crucial role in stem cell maintenance, apical meristem formation, embryonic development, and various other developmental processes. However, the identification and function of *WOX* genes have not been reported in perennial loquat. In this study, 18 *EjWOX* genes were identified in the loquat genome. Chromosomal localization analysis showed that 18 *EjWOX* genes were located on 12 of 17 chromosomes. Gene structure analysis showed that all *EjWOX* genes contain introns, of which 11 *EjWOX* genes contain untranslated regions. There are 8 pairs of segmental duplication genes and 0 pairs of tandem duplication genes in the loquat *WOX* family, suggesting that segmental duplications might be the main reason for the expansion of the loquat *WOX* family. A *WOX* transcription factor gene named *EjWUSa* was isolated from loquat. The EjWUSa protein was localized in the nucleus. Protein interactions between EjWUSa with EjWUSa and EjSTM were verified. Compared with wild-type *Arabidopsis thaliana*, the *35S::EjWUSa* transgenic *Arabidopsis* showed early flowering. Our study provides an important basis for further research on the function of *EjWOX* genes and facilitates the molecular breeding of loquat early-flowering varieties.

## Introduction

Loquat is a subtropical evergreen fruit tree of the Rosaceae family (Cao et al., 2013; Zhang et al., 2016). Compared with many fruit trees, loquat has unique characteristics of flowering in winter and fruit ripening in late spring or early summer (Jiang et al., 2019a). Previous studies have found that *EjTFL1*, *EjFRI*, *EjFT*, *EjGI*, *EjCO*, *EjFD*, *EjSOC1*, *EjLEY*, *EjSVP*, *EjAP1*, and *EjSPL* played important roles in regulating loquat flowering (Chen et al., 2020; Esumi et al., 2005; Jiang et al., 2019a, 2019b, 2019c; Reig et al., 2017; Liu et al., 2013; Zhang et al., 2019a; 2016b). However, studies of transcription factors regulating loquat flowering were still limited.

Plant flowering is regulated by both external environmental factors and internal genetic factors. At present, the understanding of angiosperm flowering relies mainly on the studies of flowering regulation in the model plant *Arabidopsis*. In *Arabidopsis*, flower bud differentiation is mainly regulated by the photoperiod pathway, vernalization pathway, gibberellin (GA) pathway, autonomous flowering pathway, heat-sensing pathway, and age pathway. In this regulatory network, about 180 genes interact to regulate *Arabidopsis* flowering (Bergonzi et al., 2013; Zhou et al., 2013). Compared with *Arabidopsis*, the studies of flowering regulation in woody plants are relatively lacking and need further research.

The *WOX* family is a class of plant-specific transcription factors (Feng et al., 2021). Its members possess 60-65 amino acid residues with the helix-loop-helix-turn-helix domain (referred to as homeodomain) and specifically bind DNA by the homeodomain to activate or depress the expression of the target gene in plants (Ikeda et al., 2009; Shafique Khan et al., 2021). *WOX* genes were divided into three separate clades, modern/WUS clade (WC), intermediate clade (IC), and ancient clade (AC) according to the time of their appearance during plant evolution (Alvarez et al., 2018). Based on phylogenetic analysis, *WOX* genes were further divided into nine subgroups (Zhang et al., 2010). In addition to the homeodomain, some WOX proteins contain three other functional domains: the acidic region (Rich in glutamic acid and aspartic acid), the WUS-box (T-L-X-L-F-P-X-X, X is an uncertain amino acid), and the EAR-like motif (X-L-X-L-X-L, X is an uncertain amino acid). The WUS-box is critical for regulating stem cell identity and floral meristem size. The acidic region is the only activation domain of the WUS proteins. In addition to the WUS-box, the EAR-like motif is also a repression domain (Ikeda et al., 2009). The *WUS* gene is the earliest gene identified in the *WOX* gene family (Xu, 2021). The negative feedback loop between WUS and CLAVATA3 (CLV3) underlies the maintenance of stem cell homeostasis in the shoot apical meristem (SAM) (Yadav, 2012; Xiao et al., 2018; Lopes et al., 2021). Previous studies have demonstrated that WUS protein regulates the expression of *CLV3* gene in the organizing center and central zone by forming homodimers with itself and heterodimers with SHOOT MERISTEMLESS (STM), respectively. In turn, CLV3 forms a signaling complex with CLAVATA1 (CLV1) and CLAVATA2 (CLV2) to regulate the expression of the *WUS* gene in the organizing center (Daum et al., 2014; Perales et al., 2016; Zhou et al., 2018; Su et al., 2020). Previous studies have demonstrated that the *WOX* genes regulated plant flowering and development (Tvorogova et al., 2021). However, genome-wide identification and functional analysis of the *WOX* genes in loquat have not been reported.

In this study, we systematically identified the *WOX* family in the loquat genome and analyzed the chromosomal localization, gene structure, conserved motifs, and basic characteristics of the loquat *WOX* family. A *WOX* transcription factor gene named *EjWUSa* was isolated from the triploid loquat ‘Wuhezaoyu’. The subcellular localization of EjWUSa was observed in *Nicotiana benthamiana* leaves. Meanwhile, the interactions between EjWUSa with EjWUSa and EjSTM were verified. Finally, we overexpressed *EjWUSa* in wild-type *Arabidopsis* for functional analysis.

## Materials and methods

### Plant materials and growth conditions

Triploid loquat ‘Wuhezaoyu’ was cultivated in the orchard of loquat resources belonging to the College of Horticulture and Landscape Architecture, Southwest University. In our previous study, the development of loquat buds was divided into nine stages (Jing et al., 2020), and from July to November 2021, the loquat flower buds at 9 stages were collected from the 12 years of triploid loquat ‘Wuhezaoyu’. After removing the superficial fluff, the loquat flower buds were immediately frozen in liquid nitrogen and stored in an ultra-low temperature refrigerator at −80°C until use.

Wild-type *Arabidopsis* was used for stable transformation of the *EjWUSa* gene. Tobacco was used in transient expression assays. Both *Arabidopsis* and tobacco were grown under long-day conditions (16 h light/8 h dark) at 22°C in a controlled environment room. *Arabidopsis* leaves for qRT-PCR were immediately frozen in liquid nitrogen and stored in an ultra-low temperature refrigerator at −80°C until use.

### Identification of the *EjWOX* genes in loquat

The Hidden Markov Model (HMM) of the Homeobox (*HOX)* superfamily (PF00046) was obtained at the Pfam website (http://pfam.xfam.org/). The WOX protein sequences in the model plant *Arabidopsis* were obtained from the TAIR database (https://www.arabidopsis.org/). The *WOX* family is one of six families in the *HOX* superfamily (Feng et al., 2021). The HMM of *HOX* superfamily was used as a query to search for candidate *EjHOX* genes in the loquat genome (The data presented in the study are deposited in NGDC repository accession No. GWHBOTF00000000). A total of 134 candidate *EjHOX* genes were identified in loquat. The conserved domain of candidate *EjHOX* genes was analyzed in NCBI (http://www.ncbi.nlm.nih.gov). As shown in **Figure S1**, 18 *EjHOX* genes and 15 *AtWOX* genes were in the same branch. Therefore, 18 *EjWOX* genes were finally identified in loquat and named according to their homology with the *AtWOX* genes (**Figure S1**; **Table S1**).

### Phylogenetic tree, multiple sequence alignment, and characterizations analysis of the WOX proteins

The apple genome file (V 3.0) was downloaded from the apple information resource (GDR, https://www.rosaceae.org) (Wang, 2021). Similar to the *EjHOX* genes, 130 candidate *MdHOX* genes were identified in the apple genome. The conserved domain of candidate *MdHOX* genes was analyzed in NCBI. As shown in **Figure S2**, 17 *MdHOX* genes and 15 *AtWOX* genes were in the same branch. Therefore, 17 *MdWOX* genes were finally identified in apple and named according to their homology with the *AtWOX* genes (**Figure S2**; **Table S2**).

The WOX protein sequences in the model plant tomato were obtained from the article published by Li et al. (2018). A phylogenetic tree including WOX proteins in loquat, apple, *Arabidopsis*, and tomato was established by MEGA software (v 11.0) with the Neighbor-joining method based on the following parameters: pairwise deletion and bootstrap analysis with 1000 replicates (Munir et al., 2016). The phylogenetic tree was imported into the iTOL website (https://itol.embl.de/) for further beautification. Multiple sequence alignment of WOX proteins in loquat were analyzed using DNAMAN software (v 9.0) with the default settings. The ExPASY database (https://web.expasy.org/protparam/) was used to forecast the characteristics of EjWOX proteins including the coding sequence length, theoretical isoelectric point (PI), molecular weight, and amino acid length (Wilkins et al., 1999).

### Chromosomal localization, gene structure, conserved motif, and synteny analysis of the *EjWOX* genes in loquat

The MEME suite (http://meme-suite.org/tools/meme) was used to identify the conserved motifs (Bailey et al., 2009). Chromosomal localization, gene structure, conserved motif, and synteny analysis of the *EjWOX* genes were visualized using TBtools software (v 1.098763) (Chen et al., 2020).

### Cloning and sequence analysis of genes and promoter

Genes and promoter sequences were obtained in the loquat genome. Gene was amplified from the cDNA of the triploid loquat ‘Wuhezaoyu’ flower buds by PCR using Phanta Max Super-Fidelity DNA polymerase (Vazyme, China). The PCR product was ligated with the pMD19-T vector. Then, the cloned product was sequenced. Finally, multiple sequence alignment was performed using DNAMAN software. Based on the same method, the promoter was isolated from the DNA of ‘Wuhezaoyu’. The specific primers were listed in **Table S3**. Then, the putative cis-acing elements on the promoter region were found in the PlantCARE database (http://bioinformatics.psb.ugent.be/webtools/plantcare/html/) (Wilkins et al., 2005). The result was visualized using TBtools software.

### Gene expression analysis with qRT-PCR

The total RNA was extracted by EASYspin Plus plant RNA extraction kit (Aidlab, China), and the cDNA was synthesized using PrimeScript™ RT reagent Kit with gDNA Eraser (TaKaRa, Japan). qRT-PCR was performed using NovoStart^®^ SYBR qPCR SuperMix plus (Novoprotein, China). The loquat *EjActin* gene and *Arabidopsis AtActin* gene were used as internal controls, with the special primers in **Table S3**. Three biological replicates were applied and data were analyzed with the 2^−ΔΔCt^ method (Livak and Schmittgen, 2001).

### Subcellular localization of EjWUSa

The coding sequence (without stop codon) of *EjWUSa* was cloned into the modified pCAMBIA1300 vector (Jing et al., 2020) with the special primers in **Table S3**. The constructed fusion vector or empty vector was transformed into *Agrobacterium strain* GV3101, respectively, and then tobacco leaves were used for transient expression. An Olympus (BX35) fluorescence fluorescence signals.

### Bimolecular fluorescence complementation assay

The coding sequence (without stop codon) of *EjWUSa* was constructed into the pXY104 vector and the special primers with restriction sites (*Sal* I and *Bam*H I) were listed in **Table S3**. The coding sequence of *EjWUSa* or *EjSTM* was constructed into the pXY106 vector and the special primers with restriction sites (*Sal* I and *Bam*H I) were listed in **Table S3**. The constructed fusion vectors or empty vectors were transformed into *Agrobacterium strain* GV3101, respectively, and then tobacco leaves were used for transient expression. An Olympus (BX35) fluorescence microscope (Tokyo, Japan) was used to observe the fluorescence signals. Three independent leaves were observed. The method was described by Liu et al. (2021).

### Firefly luciferase complementation imaging assay

The coding sequence of *EjWUSa* was constructed into the pCAMBIA-CLuc vector and the special primers with restriction sites (*Sal* I and *Bam*H I) were listed in **Table S3**. The coding sequence (without stop codon) of *EjWUSa* or *EjSTM* was constructed into the pCAMBIA-NLuc vector and the special primers with restriction sites (*Sal* I and *Bam*H I) were listed in **Table S3**. The constructed fusion vectors or empty vectors were transformed into *Agrobacterium strain* GV3101, respectively, and then tobacco leaves were used for transient expression. After 2 days of dark treatment, 1 mmol of fluorescein (Promega, USA) was applied to the injection site of tobacco leaves and dark treated for six minutes. The signals were then captured with a CCD imaging instrument (Alliance, UK). Three independent leaves were observed. The method was described by Liu et al. (2021).

### *Arabidopsis* transformation

The coding sequence of *EjWUSa* was cloned into the pFGC5941 vector with the special primers in **Table S3**. The constructed fusion vector was transformed into *Agrobacterium strain* GV3101, and then wild-type *Arabidopsis* (WT) were used for stable expression assay. The seeds of *35S::EjWUSa* transgenic *Arabidopsis* and WT were planted in the soil after treatment at 4°C for 24 h. Basta was used to screen *35S::EjWUSa* transgenic *Arabidopsis*. DNA was extracted from *35S::EjWUSa* transgenic *Arabidopsis* and WT leaves and then PCR was performed. RNA was extracted from *35S::EjWUSa* transgenic *Arabidopsis* and WT and then expression analysis was performed using special primers in **Table S3**. Finally, we obtained the T3 homozygous *35S::EjWUSa* transgenic lines. We counted the bolting time and the flowering time of *35S::EjWUSa* transgenic *Arabidopsis* and WT, respectively. Meanwhile, we counted the number of rosette leaves of *35S::EjWUSa* transgenic *Arabidopsis* and WT when the flowering shoot was 1 cm, respectively. All data were analyzed for significance by SPSS 26.0 software with One‐way ANOVAs analysis.

## Results

### Identification and phylogenetic tree of *EjWOX* genes in loquat

**Figure 1:**
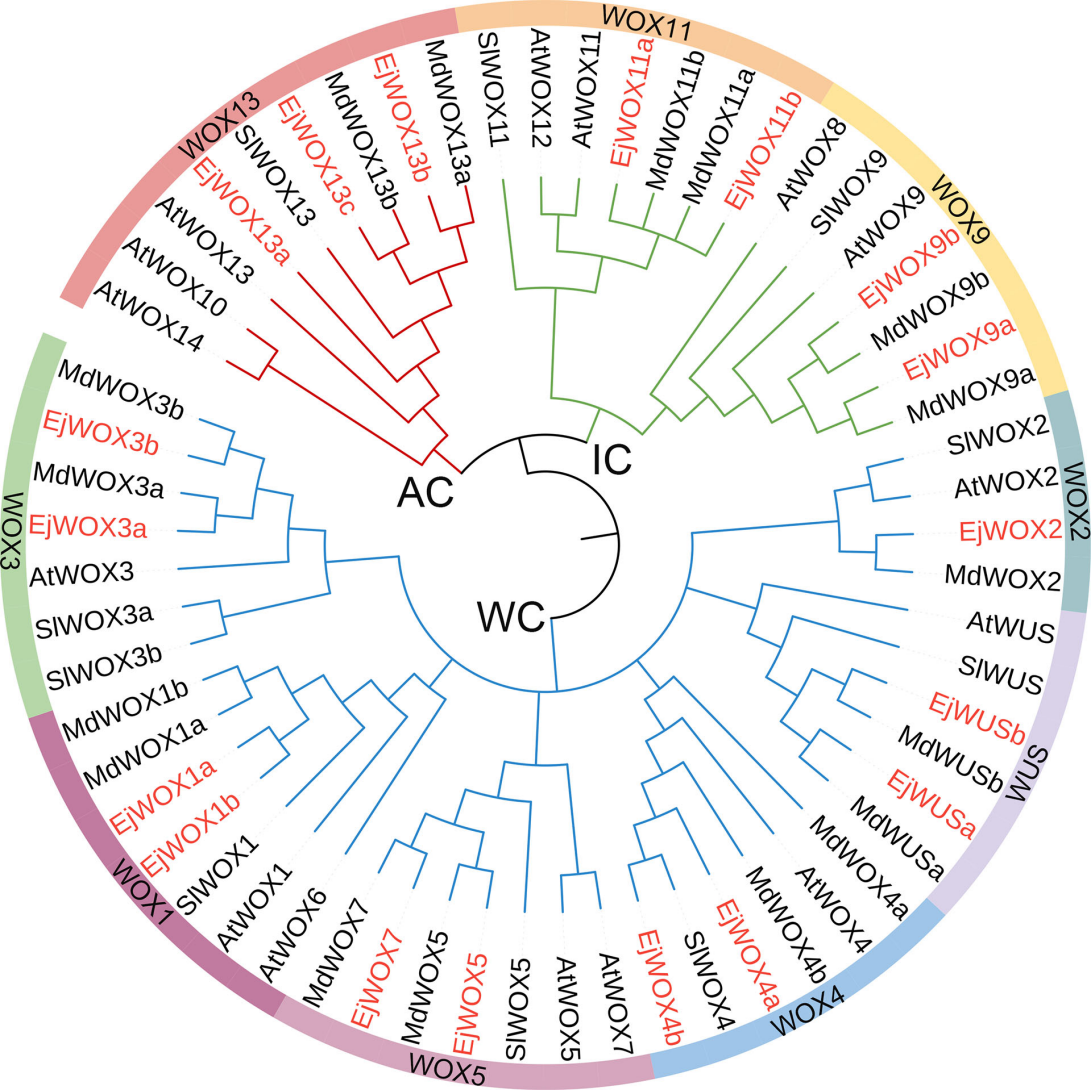
The phylogenetic tree including WOX proteins from *Eriobotrya japonica, Malus domestica*, *Solanum lycopersicum*, and *Arabidopsis thaliana.* Nine subgroups of the *WOX* family are represented by different colors. WC, WUS clade. IC, intermediate clade. AC, ancient clade. The WOX proteins in loquat are marked with red.

**Figure 2:**
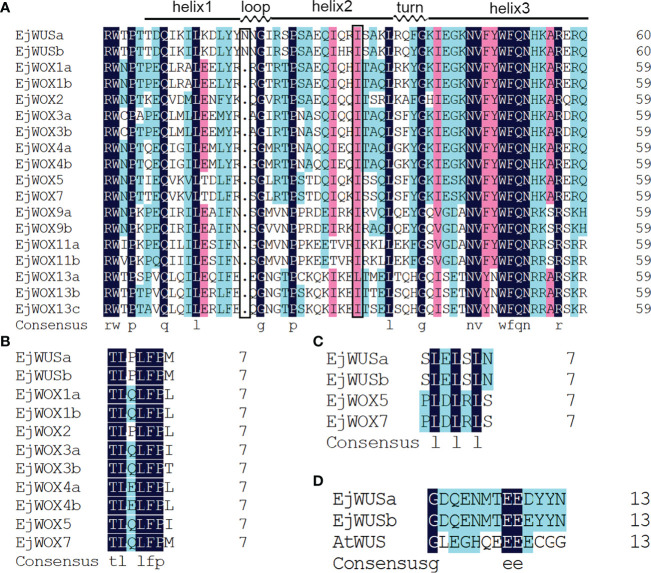
Multiple sequence alignment of WOX proteins in loquat. **(A)** Multiple sequence alignment of the homeodomain. An extra amino acid residue in the black box was observed in the homeodomain of the EjWOX proteins within the WUS subgroup. **(B)** Multiple sequence alignment of the WUS-box. **(C)** Multiple sequence alignment of the EAR-like motif. **(D)** Multiple sequence alignment of the acidic region. The identical residues are in black boxes.

The HMM of *HOX* superfamily was used as a query to search for candidate *EjHOX* genes in the loquat genome. A total of 134 candidate *EjHOX* genes were identified in the loquat genome, and the conserved domain of candidate *EjHOX* genes was analyzed in NCBI. The result showed 18 *EjWOX* genes were finally identified in the loquat (**Figure S1**). Phylogenetic analysis showed that the WOX proteins in loquat, apple, *Arabidopsis*, and tomato were similar (**Figure 1**). The *EjWOX* genes were classified into three well-supported clades (**Figure 1**). The WUS clade had the largest number of members, 11 in total, and the intermediate clade and ancient clade contained 4 and 3 members, respectively (**Figure 1**). The *EjWOX* genes were divided into nine subgroups based on phylogenetic analysis (**Figure 1**). Multiple sequence alignment showed that 13 amino acid residues are strictly conserved in the homeodomain of the EjWOX proteins, including Q and L in helix1, G in loop, P and L in helix2, G in turn, and N, V, W, F, Q, N, and R in helix3 (**Figure 2A**). An extra amino acid residue in the black box was observed in the homeodomain of the EjWOX proteins within the WUS subgroup (**Figure 2A**). The amino acid residue might be essential for their biological function. The WUS-box was found only in EjWOX proteins within the WUS clade (**Figure 2B**). The EAR-like motif was found only in EjWOX proteins within the WUS subgroup and WOX5 subgroup (**Figure 2C**). The acidic region was found only in EjWOX proteins within the WUS subgroup (**Figure 2D**).

### Chromosomal localization, synteny analysis, and characterizations of the *EjWOX* genes in loquat

**Figure 3:**
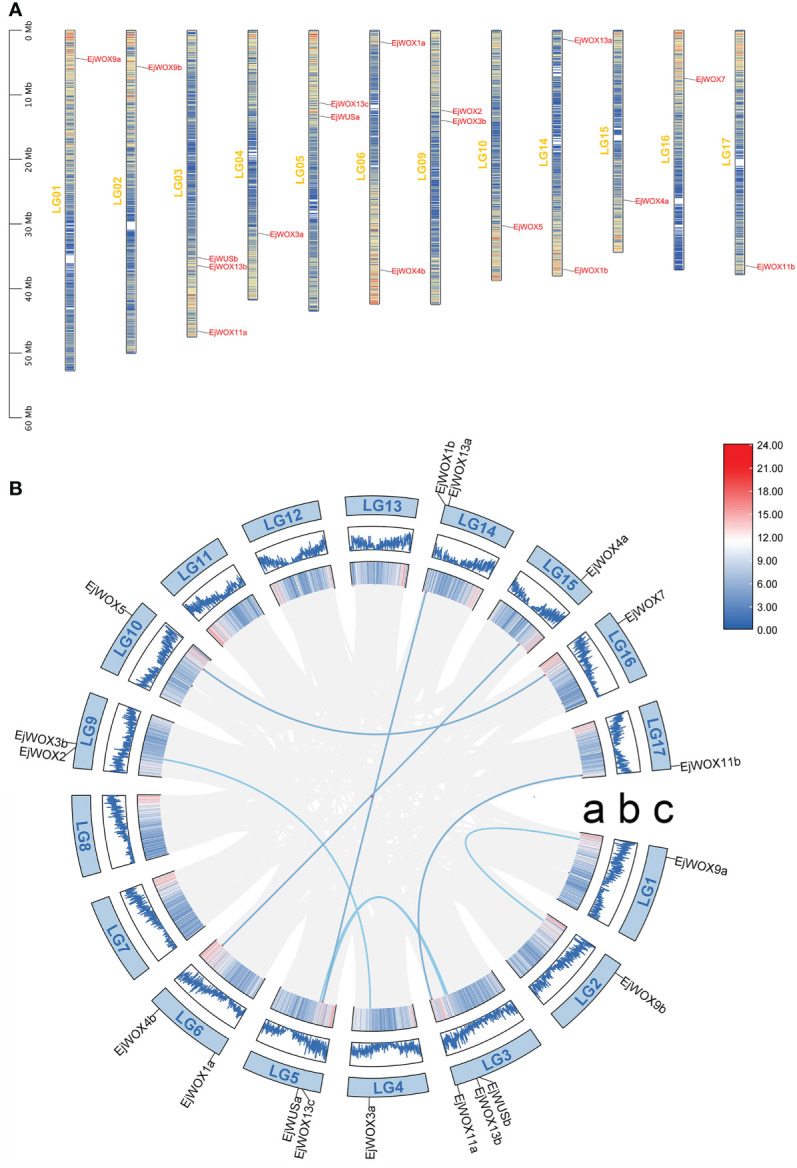
Chromosomal localization and synteny analysis of the *EjWOX* genes. **(A)** The chromosomal localization of the *EjWOX* genes. The chromosome numbers are marked on the left of each chromosome. Gene names are marked to the right of each chromosome. The black line with the scale on the left represents the length of the chromosomes. The colored lines within these columns represent gene density. **(B)** The synteny analysis of the *EjWOX* genes. Syntenic gene pairs are connected by blue lines. a: The colored lines indicate gene density. b: The width of the blue lines represents the gene density. c: LG01-LG17 represent 17 loquat chromosomes.

We analyzed the localization of 18 *EjWOX* genes on 17 loquat chromosomes. The 18 *EjWOX* genes were unevenly distributed on 12 of the 17 chromosomes (**Figure 3A**). No *EjWOX* genes were distributed on chromosomes 7, 8, 11, 12, and 13 (**Figure 3A**). Chromosomes 1, 2, 4, 10, 15, 16, and 17 had only one *EjWOX* gene (**Figure 3A**). Chromosomes 5, 6, 9, and 14 had two *EjWOX* genes (**Figure 3A**). Moreover, chromosome 3 had the highest number of *EjWOX* genes, with three *EjWOX* genes (**Figure 3A**). A total of 8 pairs of segmental duplication genes and 0 pairs of tandem duplication genes were identified in the loquat *WOX* family (**Figure 3B**). The length of EjWOX proteins ranged from 176 aa (EjWOX7) to 409 aa (EjWOX9a) with an average of 286.94 aa (**Table 1**). Their molecular weights ranged from 20.16 KDa (EjWOX7) to 45.14 KDa (EjWOX9a), with an average of 32.25 KDa (**Table 1**). In addition, these proteins might be mainly composed of basic amino acids with isoelectric points ranging from 4.71 (EjWOX13a) to 9.26 (EjWOX4b), with an average of 7.48 (**Table 1**).

**Table 1:** The characteristics of *EjWOX* genes in loquat.

Gene name	Gene locus	Chr.no.	Strand direction	Location	Protein		
					Lenth(aa)	Mol.Wt.(KDa)	pl
EjWUSa	Eja05G013550.1	5	–	10997929-10999501	328	36.06	6.67
EjWUSb	Eja03G017360.1	3	+	34685761-34687314	324	35.56	6.86
EjWOX1a	Eja06G001390.1	6	+	2303751-2306980	381	43.06	6.77
EjWOX1b	Eja14G019830.1	14	–	50634-53908	376	42.649	7.76
EjWOX2	Eja09G010840.1	9	+	10468783-10470302	271	30.57	8.81
EjWOX3a	Eja04G017350.1	4	+	29535577-29538433	244	28.15	7.71
EjWOX3b	Eja09G011670.1	9	–	11986178-11989035	237	27.28	8.97
EjWOX4a	Eja15G014170.1	15	–	27685794-27687603	239	26.73	9.26
EjWOX4b	Eja06G024530.1	6	–	37418904-37420581	238	26.75	9.24
EjWOX5	Eja10G018800.1	10	–	29983652-29984397	179	20.41	8.76
EjWOX7	Eja16G008720.1	16	+	6688246-6688915	176	20.16	8.76
EjWOX9a	Eja01G005310.1	1	–	4323964-4327524	409	45.14	7.88
EjWOX9b	Eja02G005930.1	2	–	4892997-4895908	407	45	7.19
EjWOX11a	Eja03G028280.1	3	–	46141451-46144547	302	32.85	5.48
EjWOX11b	Eja17G022460.1	17	–	36473574 36474613	274	30.6	8.45
EjWOX13a	Eja14G000850.1	14	+	1721638- 1724233	235	27.12	4.71
EjWOX13b	Eja03G018500.1	3	+	35974797- 35978213	277	31.65	5.62
EjWOX13c	Eja05G011640.1	5	–	9250942- 9253815	268	30.82	5.72

"+" and "-" represent strand direction.

### Conserved motif and gene structure analysis of the *EjWOX* genes in loquat

**Figure 4:**
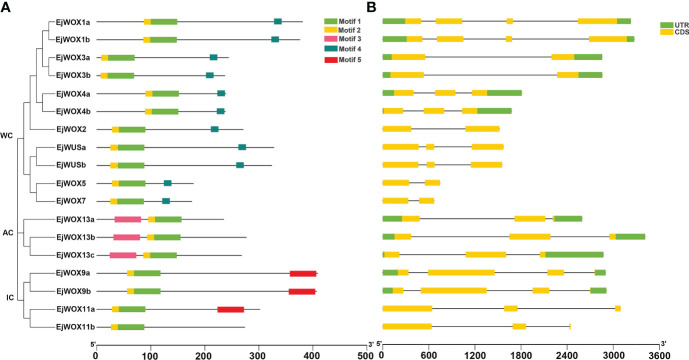
Conserved motif and gene structure analysis of loquat *WOX* genes. **(A)** Conserved motifs in EjWOX proteins are represented by colored boxes. **(B)** UTRs, exons, and introns are represented by green squares, yellow squares, and gray lines, respectively. Black lines indicate length. WC, WUS clade. IC, intermediate clade. AC, ancient clade.

The conserved motifs of the loquat WOX proteins were analyzed using MEME program and five different motifs were obtained (**Figure 4A**). All EjWOX proteins contain motif 1 and motif 2 (**Figure 4A**). Sequence analysis showed that motif 1 and motif 2 make up the homeodomain. All EjWOX proteins within the WUS clade contain motif 4 (**Figure 4A**). Sequence analysis showed that motif 4 is the WUS-box. All EjWOX proteins within the ancient clade contain motif 3 (**Figure 4A**). Except for EjWOX11b, all EjWOX proteins within the intermediate clade contain motif 5 (**Figure 4A**). EjWOX proteins within the same clade contained similar motifs, indicating that they might undertake similar biological functions (**Figure 4A**). We further analyzed the exon-intron structure of the *EjWOX* genes. All *EjWOX* genes contain introns (**Figure 4B**). The intron number of *EjWOX* genes varied from one to three (**Figure 4B**). Among the 18 *EjWOX* genes, there were 11 genes containing untranslated regions (**Figure 4B**). Only 7 *EjWOX* genes did not contain untranslated regions, i.e., *EjWUSa*, *EjWUSb*, *EjWOX2*, *EjWOX5*, *EjWOX7*, *EjWOX11a*, and *EjWOX11b* (**Figure 4B**). The number and length of exons, introns, and untranslated regions (UTRs) were conserved in *EjWOX* genes within the same subgroup (**Figure 4B**).

### Temporal expression patterns of *EjWUSa* and *EjWUSb* in loquat flower buds

**Figure 5:**
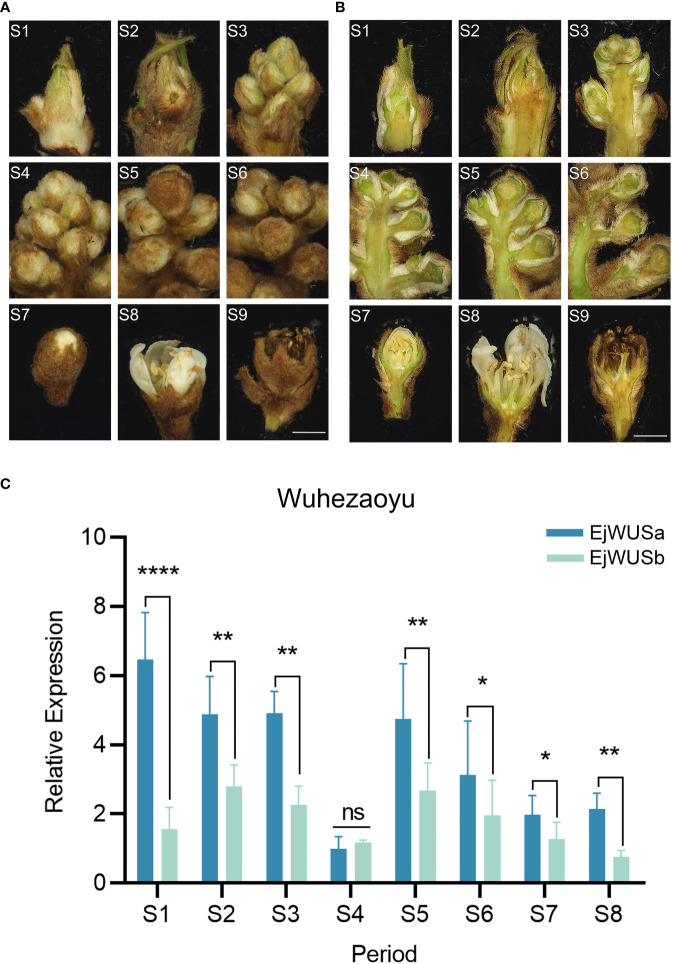
Inflorescence morphology and morphological anatomy of the triploid loquat ‘Wuhezaoyu’ and the temporal expression patterns of *EjWUSa* and *EjWUSb* in the triploid loquat ‘Wuhezaoyu’ flower buds. **(A, B)** Inflorescence morphology and morphological anatomy of the triploid loquat ‘Wuhezaoyu’. S1: Vegetative apex; S2: Floral meristem initiation and flower bud differentiation; S3: Rapid differentiation of floral buds; S4: Panicle elongation; S5: Floral bud elongation with visible floral buds; S6: Elongation of branches in a panicle; S7: White corollas of floral buds; S8: Floral anthesis and full bloom; S9: Petal fall. Bars = 2000µm. **(C)** The temporal expression patterns of *EjWUSa* and *EjWUSb* in the triploid loquat ‘Wuhezaoyu’ flower buds. *Ejactin* as an internal control. Error bars indicate Standard Error (SE) from three biological replicates. Asterisks or n.s. indicate significant differences between *EjWUSa* and *EjWUSb*, *p<0.05, **p<0,01, **** P < 0.0001, ns, no significant difference, by One-way ANOVAs.

From July to November 2021, the loquat flower buds at 9 stages were collected from the 12-year-old triploid loquat ‘Wuhezaoyu’. The RNA degradation of loquat flower buds at the petal fall was serious, so the loquat flower buds at the petal fall were not suitable for qRT-PCR (**Figure 5A, B**). Transcriptome analysis of loquat flowers showed that *WUS* might be a floral meristem identity gene (Jing et al., 2020). We analyzed the expression levels of *EjWUSa* and *EjWUSb* in loquat flowers at different stages. In the early stages of flower development, the expression level of *EjWUSa* was significantly higher than that of *EjWUSb* (**Figure 5C**), suggesting that *E*j*WUSa* might play an important role in the transition from the vegetative apex to reproductive apex. Therefore, we further cloned *EjWUSa* from loquat and then investigated its role in loquat flowering.

### Isolation of *EjWUSa* from loquat

The *EjWUSa* was isolated from the cDNA of triploid loquat ‘Wuhezaoyu’ flowers (**Figure S3**), which contains three exons and two introns, but no untranslated region (**Figure 4B**). The coding sequence of *EjWUSa* is 987 bp, encoding 328 amino acids (**Table 1**). The EjWUSa contains four conserved domains from the N-terminal to the C-terminal, followed by the homeodomain, the acidic region, the WUS-box, and the EAR-like motif (**Figure 2**). The *EjWUSa* promoter was cloned from the DNA of the triploid loquat ‘Wuhezaoyu’ leaves (**Figure S3**; **Table S4**). We further analyzed the binding elements on the *EjWUSa* promoter (**Figure S4**). The result showed that the *EjWUSa* promoter contains one CAT-box related to meristem expression (**Figure S4**) (Lin et al., 2022), suggesting that the *EjWUSa* might be involved in forming the shoot apical meristem. In addition, the *EjWUSa* promoter also contains two Box4s and two TCCC-motifs (**Figure S4**) (Chen and Qiu, 2020), indicating that the expression of *EjWUSa* might be regulated by light signals.

### Subcellular localization of EjWUSa

**Figure 6:**
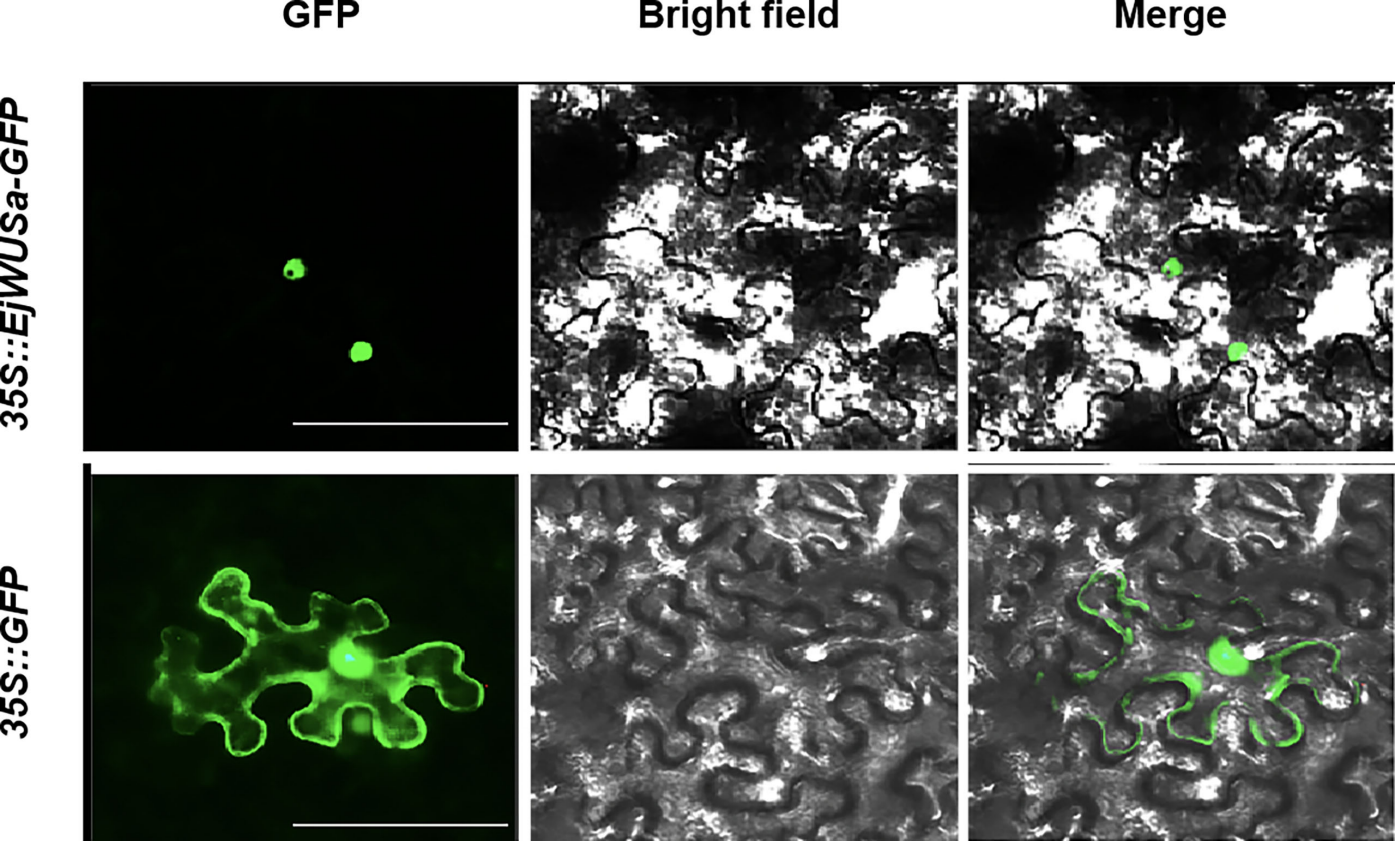
Subcellular localization of EjWUSa in tobacco leaves. GFP, GFP fluorescence channel; Merged, merged image of GFP and bright field. Bars = 50 µm.

The coding sequence (without stop codon) of *EjWUSa* was cloned into the pCAMBIA1300 vector to generate a fusion protein in tobacco cell. The green fluorescent protein (GFP) in the control group was localized in the nucleus and cell membrane, while the EjWUSa-GFP fusion protein in the experimental group was localized in the nucleus (**Figure 6**). It showed that the EjWUSa protein was localized in the nucleus, consistent with the characteristics of transcription factors.

### The interactions between EjWUSa with EjWUSa and EjSTM

**Figure 7:**
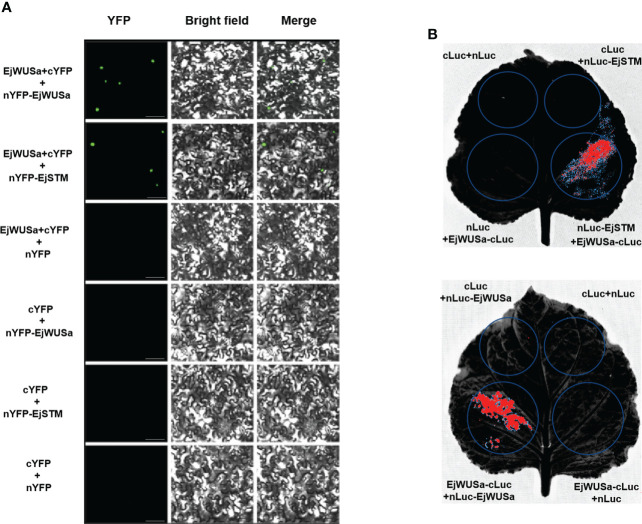
Protein interactions between EjWUSa with EjWUSa and EjSTM. **(A)** Protein interactions between EjWUSa with EjWUSa and EjSTM were demonstrated in the BIFC assay. The scale bar is 20 μm. **(B)** Protein interactions between EjWUSa with EjWUSa and EjSTM were demonstrated in the LCI assay.

To investigate whether the protein interactions between WUS and WUS and STM are conserved across species, we isolated the *EjSTM* from the cDNA of the triploid loquat ‘Wuhezaoyu’ flower buds (**Figure S3**; **Table S5**). The BiFC assay and LCI assay were used to verify whether EjWUSa can form a dimer with EjSTM or EjWUSa in tobacco cells. In the experimental groups, strong signals were observed in tobacco cells, while no signals were observed in the control groups (**Figure 7**). This suggested that EjWUSa formed dimers with EjWUSa or EjSTM, respectively, which provided the basis for the formation of a complete YFP or LUC.

### *Arabidopsis* transformation

**Figure 8:**
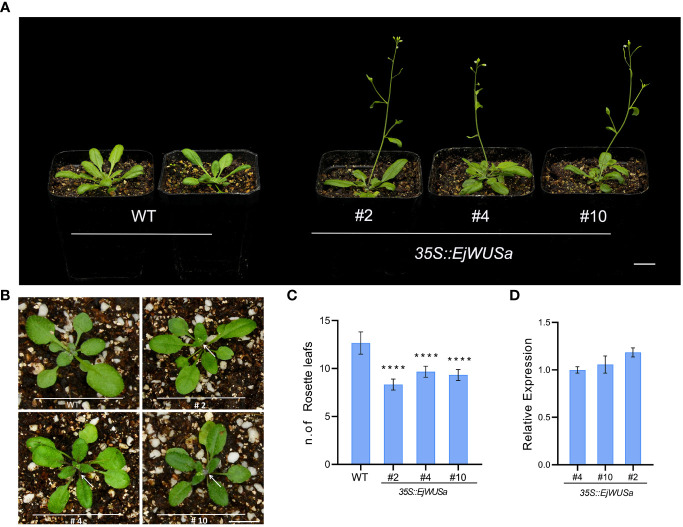
Overexpression of *EjWUSa* promotes *Arabidopsis* flowering. **(A)** *35S::EjWUSa* transgenic lines showed early flowering compared to WT. Bars = 1cm. **(B)** *35S::EjWUSa* transgenic lines showed early bolting compared to WT. Bars = 1cm. **(C)** The number of rosette leaves of *35S::EjWUSa* transgenic lines and WT while blooming. **(D)** The expression level of *EjWUSa* in *35S::EjWUSa* transgenic lines and WT. The leaves of *35S::EjWUSa* transgenic *Arabidopsis* and WT were collected after flowering and used for qRT-PCR analysis. Error bars indicate SE from three biological replicates. Asterisks indicate significant differences between *35S::EjWUSa* transgenic lines and WT, ****p < 0.0001, by One-way ANOVAs.

To investigate the function of *EjWUSa*, an overexpression vector containing the coding sequence of the *EjWUSa* gene was constructed and transformed into wild-type *Arabidopsis* (WT). After Basta screening and PCR identification, we obtained T3 homozygous *35S::EjWUSa* transgenic lines (**Figure S5**). The expression level of *EjWUSa* in *35S::EjWUSa* transgenic lines was higher than in WT (**Figure 8D**). Under the same growing conditions, WT had about 12 rosette leaves, whereas the *35S::EjWUSa* transgenic lines had only seven to nine rosette leaves (**Figures 8B, C**). All *35S::EjWUSa* transgenic lines had approximately 10 days early bolting and 9 days early flowering compared to WT (**Figures 8A, B**). In conclusion, all *35S::EjWUSa* transgenic lines showed an early flowering phenotype compared to WT. Compared to WT, the *35S::EjWUSa* transgenic lines had no significant difference in morphological characteristics such as flower organs, leaf shape, siliques, stems, and leaves. From the above results, *EjWUSa* has the function of promoting *Arabidopsis* flowering.

## Discussion

As a category of transcription factors regulating stem cell fate, WOX proteins are involved in many physiological processes related to plant growth and development (van der Graaff et al., 2009; Gambino et al., 2011; Lin et al., 2012; Kanchan and Sembi, 2020). Due to advances in omics technologies, genome-wide identification of the *WOX* gene family has been accomplished in several species, including *Arabidopsis*, sorghum, maize, tobacco, potato, walnut, and sweet orange. These plants contain 15, 11, 21, 10, 8, 14, and 8 *WOX* genes, respectively (2020; Vandenbussche et al., 2009; Zhang et al., 2010; Li et al., 2018; Shafique Khan et al., 2021). However, genome-wide identification and function of the *WOX* genes have not been reported in loquat. In the present study, we performed the genome-wide identification of the *WOX* gene family in loquat. Meanwhile, we further investigated the role of *EjWUSa* in loquat flowering.

A total of 18 *EjWOX* genes were identified in the loquat genome (**Table S1**). Like the *AtWOX* genes, the *EjWOX* genes can be divided into three clades or nine subgroups. Nevertheless, the homologs of *Arabidopsis AtWOX6*, *AtWOX10*, and *AtWOX14* could not be found in loquat (**Figure 1**). The substitutability of *WO*X genes might make their loss possible (Feng et al., 2021). Gene duplication is the main reason for gene family expansion (Feng et al., 2021). In our study, 8 pairs of segmental duplication genes and 0 pairs of tandem duplication genes were identified in the loquat *WOX* family (**Figure 3B**), suggesting that segmental duplications might be the main reason for the expansion of the loquat *WOX* family. The homeodomain is conserved in the *WOX* gene family among different species and maintains the functional integrity of *WOX* genes (Zhang et al., 2010; Kanchan and Sembi, 2020). As shown in **Figure 2A**, the 11th amino acid residue (In the black box) in helix 2 of the homeodomain is not strictly conserved in loquat WOX proteins, which differs from that of other species, such as apple (**Figure S6**), walnut, tomato, potato, rice, sorghum, maize, *Arabidopsis*, and poplar (Zhang et al., 2010; Li et al., 2018; Chang et al., 2019). We found a fragment of the EjWUS proteins consisting of 10 amino acids residues, rich in glutamate residues and aspartate residues, and with the same number of acidic residues as the AtWUS protein (**Figure 2D**). It should be considered as an acidic region. Therefore, the acidic region is not a conserved domain specific to the AtWUS protein. However, the acidic region was not detectable in WUS proteins in rice, sorghum, or maize (Zhang et al., 2010). The *EjWOX* genes within the ancient clade were more conserved in the number and length of exons, introns, and UTRs compared to those within the intermediate and modern clades, consistent with previous studies (**Figure 4B**) (Deveaux et al., 2008).

EjWUSa was shown to interact with EjWUSa and EjSTM (**Figure 7**), consistent with studies in *Arabidopsis* (Perales et al., 2016; Zhou et al., 2018). This suggested that protein interactions between WUS with WUS and STM might be conserved across species. In *Arabidopsis*, AtWOX proteins were essential for embryonic patterning, stem cell maintenance, and organ formation (Ji et al., 2010). *AtWOX2* was involved in the formation of apical-basal axis (2008; Palovaara, 2010). The *wox3/prs1* mutant *Arabidopsis* exhibited short stature, abnormal sepal number and morphology, and the absence of stamens and stipules (Shimizu et al., 2009). *OsWOX3A*, which is the rice homolog of *AtWOX3*, was involved in the pleiotropic effects of organ development (Cho et al., 2013). As a homolog of *AtWOX9, Cymose Petunia WOX9* played an important role in the inflorescence development (Rebocho et al., 2008). *AtWOX13* and *AtWOX14* played important roles in the development of roots and the formation of floral organs (Deveaux et al., 2008). *AtWOX5* maintained the maintenance of stem cell homeostasis in the root apical meristem (RAM) (Sarkar et al., 2007; Nardmann et al., 2009; Tian et al., 2014). In *Arabidopsis* shoot and floral meristems, the *AtWUS* gene was required for stem cell identity (Schoof et al., 2000; Nardmann et al., 2007). When the *AtWUS* gene was mutated, it caused an early termination of inflorescence meristem (Laux et al., 1996). *Arabidopsis* overexpressing the *AtWUS* gene showed ectopic flower bud growth and callus-like formation (Xu et al., 2005). Functional verification of the *WUS* gene in other plants has been reported. For example, overexpression of the *GhWUS* gene increased the embryogenic callus formation in *Gossypium hirsutum* (Xiao et al., 2018). WUS protein has anti-virus activity by repressing MTase expression (Xu, 2021). In this study, *35S::EjWUSa* transgenic *Arabidopsis* exhibits early flowering (**Figure 8**). Unlike studies in *Arabidopsis*, we did not find the callus-like tissue on the stems of transgenic lines. Multiple sequence alignment showed that the similarity between EjWUSa and AtWUS was only 32.94%, which might lead to their functional differences (**Figure S7**). This also reflects the difference in genetic information between woody plants and herbs.

## Conclusions

We reported the first genome-wide identification of the *WOX* family in perennial loquat. In the present study, 18 *EjWOX* genes were identified in the loquat genome. We further analyzed the evolutionary features and basic characteristics of the loquat *WOX* genes. A total of 8 pairs of segmental duplication genes and 0 pairs of tandem duplication genes were identified in the loquat *WOX* family, suggesting that segmental duplications might be the main reason for the expansion of the loquat *WOX* family. Compared with WT, *35S::EjWUSa* transgenic *Arabidopsis* exhibits early flowering. Our study provides an important basis for further research on the function of *EjWOX* genes in the future and also facilitates the molecular breeding of loquat early-flowering varieties.

## Data availability statement

The original contributions presented in the study are publicly available. This data can be found here: https://ngdc.cncb.ac.cn/gwh, GWHBOTF00000000.

## Author contributions

YY and MY performed the experiments and drafted the manuscript. XL contributed to morphology analysis of diploid loquat. YX, RH, and QX contributed to the data analysis. QG, DJ, and YX provided plant tissues, laboratory facilities, and project supervision. All authors approved the final draft of the manuscript.

## Funding

This work was financially supported by The National Natural Science Foundation of China (Grant Numbers: 32102321), the National Key R&D Program of China (No. 2019YFD1000900), the Chongqing Science and Technology Commission (cstc2021jscx-gksbX0010 and cstc2021jcyj-msxmX1156), the Innovation Research Group Funds for Chongqing Universities (CXQT19005), the Chongqing Forestry Administration (YuLinKeYan2022-14), and the Characteristic Fruit Industry and Technology System Innovation Team of Chongqing Agriculture and Rural Affairs Commission: No.2020[3]01.

## Conflict of interest

The authors declare that the research was conducted in the absence of any commercial or financial relationships that could be construed as a potential conflict of interest.

## Publisher’s note

All claims expressed in this article are solely those of the authors and do not necessarily represent those of their affiliated organizations, or those of the publisher, the editors and the reviewers. Any product that may be evaluated in this article, or claim that may be made by its manufacturer, is not guaranteed or endorsed by the publisher.

## References

[B1] Alvarez J. M. Bueno N. Cañas R. A. Avila C. Cánovas F. M. Ordás R. J. (2018). Analysis of the WUSCHEL-RELATED HOMEOBOX gene family in *Pinus pinaster*: New insights into the gene family evolution. Plant Physiol. Biochem. 123, 304–318. doi: 10.1016/j.plaphy.2017.12.031 29278847

[B2] Bailey T. L. Boden M. Buske F. A. Frith M. Grant C. E. Clementi L.. (2009). MEME SUITE: tools for motif discovery and searching. Nucleic Acids Res. 37 (Web Server issue), W202–W208. doi: 10.1093/nar/gkp335 19458158 PMC2703892

[B3] Bergonzi S. Albani M. C. Ver Loren van Themaat E. Nordström K. J. V. Wang R. Schneeberger K.. (2013). Mechanisms of age-dependent response to winter temperature in perennial flowering of *Arabis alpina* . Sci. (New York N.Y.) 340 (6136), 1094–1097. doi: 10.1126/science.1234116 23723236

[B4] Cao S. Yang Z. Zheng Y. (2013). Sugar metabolism in relation to chilling tolerance of loquat fruit. Food Chem. 136 (1), 139–143. doi: 10.1016/j.foodchem.2012.07.113 23017404

[B5] Chang Y. Song X. Zhang Q. Liu H. Bai Y. Lei X.. (2019). Genome-wide identification of WOX gene family and expression analysis during rejuvenational rhizogenesis in walnut (*Juglans regia* l.). Forests 11 (1), 16. doi: 10.3390/f11010016

[B6] Chen C. Chen H. Zhang Y. Thomas H. R. Frank M. H. He Y.. (2020). TBtools: an integrative toolkit developed for interactive analyses of big biological data. Mol. Plant 13 (8), 1194–1202. doi: 10.1016/j.molp.2020.06.009 32585190

[B7] Chen S. Qiu G. (2020). Cloning and activity analysis of the promoter of nucleotide exchange factor gene ZjFes1 from the seagrasses *Zostera japonica* . Sci. Rep. 10 (1), 17291. doi: 10.1038/s41598-020-74381-6 33057160 PMC7560745

[B8] Chen W. Wang P. Wang D. Shi M. Xia Y. He Q.. (2020). EjFRI, FRIGIDA (FRI) ortholog from *Eriobotrya japonica*, delays flowering in *Arabidopsis* . Int. J. Mol. Sci. 21 (3), E1087. doi: 10.3390/ijms21031087 32041257 PMC7038142

[B9] Cho S.-H. Yoo S.-C. Zhang H. Pandeya D. Koh H.-J. Hwang J.-Y.. (2013). The rice narrow leaf2 and narrow leaf3 loci encode WUSCHEL-related homeobox 3A (OsWOX3A) and function in leaf, spikelet, tiller and lateral root development. New Phytol. 198 (4), 1071–1084. doi: 10.1111/nph.12231 23551229

[B10] Daum G. Medzihradszky A. Suzaki T. Lohmann J. U. (2014). A mechanistic framework for noncell autonomous stem cell induction in *Arabidopsis* . Proc. Natl. Acad. Sci. U. S. A. 111 (40), 14619–14624. doi: 10.1073/pnas.1406446111 25246576 PMC4210042

[B11] Deveaux Y. Toffano-Nioche C. Claisse G. Thareau V. Morin H. Laufs P.. (2008). Genes of the most conserved WOX clade in plants affect root and flower development in *Arabidopsis* . BMC Evol. Biol. 8, 291. doi: 10.1186/1471-2148-8-291 18950478 PMC2584047

[B12] Esumi T. Tao R. Yonemori K. (2005). Isolation of LEAFY and TERMINAL FLOWER 1 homologues from six fruit tree species in the subfamily maloideae of the rosaceae. Sex. Plant Reprod. 17 (6), 277–287. doi: 10.1007/s00497-004-0239-3

[B13] Feng C. Zou S. Gao P. Wang Z. (2021). In silico identification, characterization expression profile of WUSCHEL-related homeobox (WOX) gene family in two species of kiwifruit. PeerJ 9, e12348. doi: 10.7717/peerj.12348 34760371 PMC8557698

[B14] Gambino G. Minuto M. Boccacci P. Perrone I. Vallania R. Gribaudo I. (2011). Characterization of expression dynamics of WOX homeodomain transcription factors during somatic embryogenesis in *Vitis vinifera* . J. Exp. Bot. 62 (3), 1089–1101. doi: 10.1093/jxb/erq349 21127025

[B15] Ikeda M. Mitsuda N. Ohme-Takagi M. (2009). *Arabidopsis* WUSCHEL is a bifunctional transcription factor that acts as a repressor in stem cell regulation and as an activator in floral patterning. Plant Cell 21 (11), 3493–3505. doi: 10.1105/tpc.109.069997 19897670 PMC2798335

[B16] Jiang Y. Peng J. Wang M. Su W. Gan X. Jing Y.. (2019a). The role of EjSPL3, EjSPL4, EjSPL5, and EjSPL9 in regulating flowering in loquat (*Eriobotrya japonica* lindl.). Int. J. Mol. Sci. 21 (1), E248. doi: 10.3390/ijms21010248 31905863 PMC6981807

[B17] Jiang Y. Peng J. Zhang Z. Lin S. Lin S. Yang X. (2019b). The role of EjSVPs in flower initiation in *Eriobotrya japonica* . Int. J. Mol. Sci. 20 (23), E5933. doi: 10.3390/ijms20235933 31779080 PMC6928820

[B18] Jiang Y. Peng J. Zhu Y. Su W. Zhang L. Jing Y.. (2019c). The role of EjSOC1s in flower initiation in *Eriobotrya japonica* . Front. Plant Sci. 10. doi: 10.3389/fpls.2019.00253 PMC6409497 30930912

[B19] Jing D. Chen W. Hu R. Zhang Y. Xia Y. Wang S.. (2020). An integrative analysis of transcriptome, proteome and hormones reveals key differentially expressed genes and metabolic pathways involved in flower development in loquat. Int. J. Mol. Sci. 21 (14), E5107. doi: 10.3390/ijms21145107 32698310 PMC7404296

[B20] Jing D. Chen W. Xia Y. Shi M. Wang P. Wang S.. (2020). Homeotic transformation from stamen to petal in *Eriobotrya japonica* is associated with hormone signal transduction and reduction of the transcriptional activity of EjAG. Physiol. Plant. 168 (4), 893–908. doi: 10.1111/ppl.13029 31587280

[B21] Ji J. Shimizu R. Sinha N. Scanlon M. J. (2010). Analyses of WOX4 transgenics provide further evidence for the evolution of the WOX gene family during the regulation of diverse stem cell functions. Plant Signa Behav. 5 (7), 916–920. doi: 10.4161/psb.5.7.12104 PMC3014546 20495368

[B22] Kanchan M. Sembi J. (2020). Comparative transcriptomic analyses of four phalaenopsis species to identify and characterize the WUSCHEL -related homeobox (WOX ) gene family. Biotechnologia 101 (4), 309–322. doi: 10.5114/bta.2020.100423

[B23] Laux T. Mayer K. F. Berger J. Jürgens G. (1996). The WUSCHEL gene is required for shoot and floral meristem integrity in *Arabidopsis* . Dev. (Cambridge England) 122 (1), 87–96. doi: 10.1242/dev.122.1.87 8565856

[B24] Li X. Hamyat M. Liu C. Ahmad S. Gao X. Guo C.. (2018). Identification and characterization of the WOX family genes in five solanaceae species reveal their conserved roles in peptide signaling. Genes 9 (5), E260. doi: 10.3390/genes9050260 29772825 PMC5977200

[B25] Li Z. Liu D. Xia Y. Li Z. Jing D. Du J.. (2020). Identification of the WUSCHEL-related homeobox (WOX) gene family, and interaction and functional analysis of TaWOX9 and TaWUS in wheat. Int. J. Mol. Sci. 21 (5), E1581. doi: 10.3390/ijms21051581 32111029 PMC7084607

[B26] Lin H. Niu L. McHale N. A. Ohme-Takagi M. Mysore K. S. Tadege M. (2012). Evolutionarily conserved repressive activity of WOX proteins mediates leaf blade outgrowth and floral organ development in plants. Proc. Natl. Acad. Sci. U. S. A. 110 (1), 366–371. doi: 10.1073/pnas.1215376110 23248305 PMC3538250

[B27] Lin M. Yan J. Ali M. M. Wang S. Tian S. Chen F.. (2022). Isolation and functional characterization of a green-tissue promoter in japonica rice (*Oryza sativa* subsp. *Japonica*). Biology 11 (8), 1092. doi: 10.3390/biology11081092 35892948 PMC9332004

[B28] Liu Y. Song H. Liu Z. Hu G. Lin S. (2013). Molecular characterization of loquat EjAP1 gene in relation to flowering. Plant Growth Regul. 70 (3), 287–296. doi: 10.1007/s10725-013-9800-0

[B29] Liu Y. Wen L. Shi Y. Su D. Lu W. Cheng Y.. (2021). Stress-responsive tomato gene SlGRAS4 function in drought stress and abscisic acid signaling. Plant Sci. 304, 110804. doi: 10.1016/j.plantsci.2020.110804 33568303

[B30] Livak K. J. Schmittgen T. D. (2001). Analysis of relative gene expression data using real-time quantitative PCR and the 2(-delta delta C(T)) method. Methods (San Diego Calif.) 25 (4), 402–408. doi: 10.1006/meth.2001.1262 11846609

[B31] Lopes F. L. Galvan-Ampudia C. Landrein B. (2021). WUSCHEL in the shoot apical meristem: old player, new tricks. J. Exp. Bot. 72 (5), 1527–1535. doi: 10.1093/jxb/eraa572 33332559

[B32] Munir S. Khan M. R. G. Song J. Munir S. Zhang Y. Ye Z.. (2016). Genome-wide identification, characterization and expression analysis of calmodulin-like (CML) proteins in tomato (*Solanum lycopersicum*). Plant Physiol. Biochem. 102, 167–179. doi: 10.1016/j.plaphy.2016.02.020 26949025

[B33] Nardmann J. Reisewitz P. Werr W. (2009). Discrete shoot and root stem cell-promoting WUS/WOX5 functions are an evolutionary innovation of angiosperms. Mol. Biol. Evol. 26 (8), 1745–1755. doi: 10.1093/molbev/msp084 19387013

[B34] Nardmann J. Zimmermann R. Durantini D. Kranz E. Werr W. (2007). WOX gene phylogeny in poaceae: A comparative approach addressing leaf and embryo development. Mol. Biol. Evol. 24 (11), 2474–2484. doi: 10.1093/molbev/msm182 17768306

[B35] Palovaara J. (2008). Conifer WOX-related homeodomain transcription factors, developmental consideration and expression dynamic of WOX2 during *Picea abies* somatic embryogenesis. Plant Mol. Biol. 66 (5), 533–549. doi: 10.1007/s11103-008-9289-5 18209956

[B36] Palovaara J. (2010). Comparative expression pattern analysis of WUSCHEL-related homeobox 2 (WOX2) and WOX8/9 in developing seeds and somatic embryos of the gymnosperm *Picea abies* . New Phytol. 188 (1), 122–135. doi: 10.1111/j.1469-8137.2010.03336.x 20561212

[B37] Perales M. Rodriguez K. Snipes S. Yadav R. K. Diaz-Mendoza M. Reddy G. V. (2016). Threshold-dependent transcriptional discrimination underlies stem cell homeostasis. Proc. Natl. Acad. Sci. U. S. A. 113 (41), E6298–E6306. doi: 10.1073/pnas.1607669113 27671653 PMC5068294

[B38] Rebocho A. B. Bliek M. Kusters E. Castel R. Procissi A. Roobeek I.. (2008). Role of EVERGREEN in the development of the cymose petunia inflorescence. Dev. Cell 15 (3), 437–447. doi: 10.1016/j.devcel.2008.08.007 18804438

[B39] Reig C. Gil-Muñoz F. Vera-Sirera F. García-Lorca A. Martínez-Fuentes A. Mesejo C.. (2017). Bud sprouting and floral induction and expression of FT in loquat [*Eriobotrya japonica* (Thunb.) lindl.]. Planta 246 (5), 915–925. doi: 10.1007/s00425-017-2740-6 28710586

[B40] Sarkar A. K. Luijten M. Miyashima S. Lenhard M. Hashimoto T. Nakajima K.. (2007). Conserved factors regulate signalling in *Arabidopsis thaliana* shoot and root stem cell organizers. Nature 446 (7137), 811–814. doi: 10.1038/nature05703 17429400

[B41] Schoof H. Lenhard M. Haecker A. Mayer K. F. Jürgens G. Laux T. (2000). The stem cell population of *Arabidopsis* shoot meristems in maintained by a regulatory loop between the CLAVATA and WUSCHEL genes. Cell 100 (6), 635–644. doi: 10.1016/s0092-8674(00)80700-x 10761929

[B42] Shafique Khan F. Zeng R.-F. Gan Z.-M. Zhang J.-Z. Hu C.-G. (2021). Genome-wide identification and expression profiling of the WOX gene family in *Citrus sinensis* and functional analysis of a CsWUS member. Int. J. Mol. Sci. 22 (9), 4919. doi: 10.3390/ijms22094919 34066408 PMC8124563

[B43] Shimizu R. Ji J. Kelsey E. Ohtsu K. Schnable P. S. Scanlon M. J. (2009). Tissue specificity and evolution of meristematic WOX3 function. Plant Physiol. 149 (2), 841–850. doi: 10.1104/pp.108.130765 19073779 PMC2633815

[B44] Su Y. H. Zhou C. Li Y. J. Yu Y. Tang L. P. Zhang W. J.. (2020). Integration of pluripotency pathways regulates stem cell maintenance in the *Arabidopsis* shoot meristem. Proc. Natl. Acad. Sci. U. S. A. 117 (36), 22561–22571. doi: 10.1073/pnas.2015248117 32839309 PMC7486707

[B45] Tian H. Wabnik K. Niu T. Li H. Yu Q. Pollmann S.. (2014). WOX5-IAA17 feedback circuit-mediated cellular auxin response is crucial for the patterning of root stem cell niches in *Arabidopsis* . Mol. Plant 7 (2), 277–289. doi: 10.1093/mp/sst118 23939433

[B46] Tvorogova V. E. Krasnoperova E. Y. Potsenkovskaia E. A. Kudriashov A. A. Dodueva I. E. Lutova L. A. (2021). [What does the WOX say? review of pegulators, targets, partners]. Mol. Biol. 55 (3), 362–391. doi: 10.31857/S0026898421030174 34097673

[B47] Vandenbussche M. Horstman A. Zethof J. Koes R. Rijpkema A. S. Gerats T. (2009). Differential recruitment of WOX transcription factors for lateral development and organ fusion in petunia and *Arabidopsis* . Plant Cell 21 (8), 2269–2283. doi: 10.1105/tpc.109.065862 19717616 PMC2751957

[B48] van der Graaff E. Laux T. Rensing S. A. (2009). The WUS homeobox-containing (WOX) protein family. Genome Biol. 10 (12), 248. doi: 10.1186/gb-2009-10-12-248 20067590 PMC2812940

[B49] Wang Y. (2021). A draft genome, resequencing, and metabolomes reveal the genetic background and molecular basis of the nutritional and medicinal properties of loquat (*Eriobotrya japonica* (Thunb.) lindl). Hortic. Res. 8 (1), 231. doi: 10.1038/s41438-021-00657-1 34719689 PMC8558328

[B50] Wilkins M. R. Gasteiger E. Bairoch A. Sanchez J. C. Williams K. L. Appel R. D.. (1999). Protein identification and analysis tools in the ExPASy server. Methods Mol. Biol. (Clifton N.J.) 112, 531–552. doi: 10.1385/1-59259-584-7:531 10027275

[B51] Xiao Y. Chen Y. Ding Y. Wu J. Wang P. Yu Y.. (2018). Effects of GhWUS from upland cotton (*Gossypium hirsutum* l.) on somatic embryogenesis and shoot regeneration. Plant Sci. 270, 157–165. doi: 10.1016/j.plantsci.2018.02.018 29576069

[B52] Xu L. (2021). WUSCHEL: The versatile protein in the shoot apical meristem. Sci. China Life Sci. 64 (1), 177–178. doi: 10.1007/s11427-020-1870-4 33373029

[B53] Xu Y.-Y. Wang X.-M. Li J. Li J.-H. Wu J.-S. Walker J. C.. (2005). Activation of the WUS gene induces ectopic initiation of floral meristems on mature stem surface in *Arabidopsis thaliana* . Plant Mol. Biol. 57 (6), 773–784. doi: 10.1007/s11103-005-0952-9 15952065

[B54] Yadav R. K. (2012). WUSCHEL protein movement and stem cell homeostasis. Plant Signaling Behav. 7 (5), 592–594. doi: 10.4161/psb.19793 PMC3419026 22516820

[B55] Zhang L. Jiang Y. Zhu Y. Su W. Long T. Huang T.. (2019). Functional characterization of GI and CO homologs from *Eriobotrya deflexa* nakai forma *koshunensis* . Plant Cell Rep. 38 (5), 533–543. doi: 10.1007/s00299-019-02384-3 30725169

[B56] Zhang L. Yu H. Lin S. Gao Y. (2016). Molecular characterization of FT and FD homologs from *Eriobotrya deflexa* nakai forma *koshunensis* . Front. Plant Sci. 7. doi: 10.3389/fpls.2016.00008 PMC4722113 26834775

[B57] Zhang X. Zong J. Liu J. Yin J. Zhang D. (2010). Genome-wide analysis of WOX gene family in rice, sorghum, maize, *Arabidopsis* and poplar. J. Integr. Plant Biol. 52 (11), 1016–1026. doi: 10.1111/j.1744-7909.2010.00982.x 20977659

[B58] Zhou Y. Yan A. Han H. Li T. Geng Y. Liu X.. (2018). HAIRY MERISTEM with WUSCHEL confines CLAVATA3 expression to the outer apical meristem layers. Sci. (New York N.Y.) 361 (6401), 502–506. doi: 10.1126/science.aar8638 PMC6095697 30072538

[B59] Zhou C.-M. Zhang T.-Q. Wang X. Yu S. Lian H. Tang H.. (2013). Molecular basis of age-dependent vernalization in *Cardamine flexuosa* . Sci. (New York N.Y.) 340 (6136), 1097–1100. doi: 10.1126/science.1234340 23723237

