# Supplementary material for: Genome-wide analysis of the WOX gene family and the role of EjWUSa in regulating flowering in loquat (Eriobotrya japonica)
Source: Front Plant Sci. 2022 Nov 3;13:1024515. doi: 10.3389/fpls.2022.1024515 (PMC9669421; doi:10.3389/fpls.2022.1024515)
Supplement: Supplementary file 2 [file DataSheet_2.docx]

**Supplementary Figures**


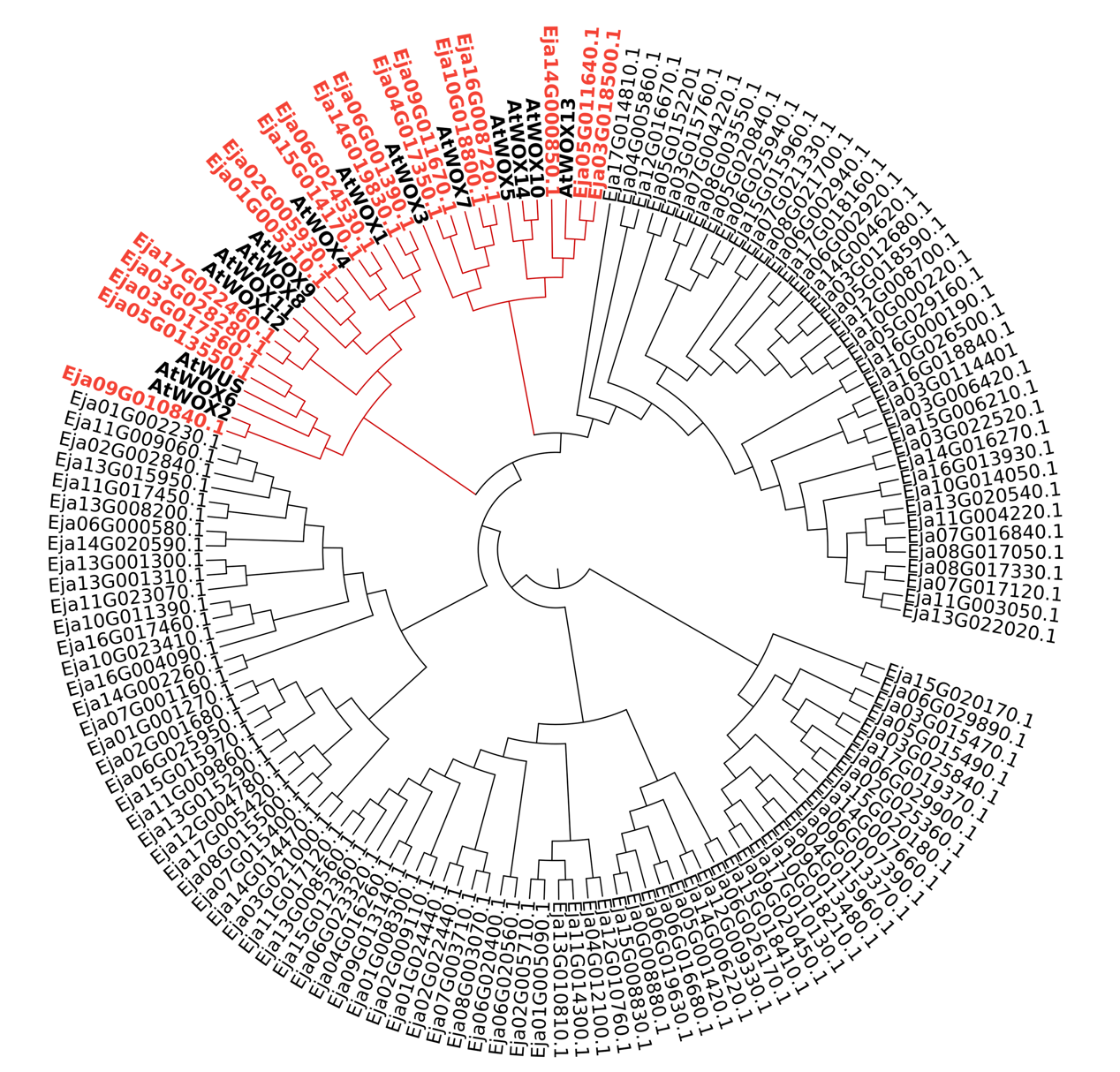


Fig. S1 The phylogenetic tree including loquat EjHOX proteins and *Arabidopsis* WOX proteins*.* 18 *EjHOX* genes were marked in red and 15 *AtWOX* genes were in the same branch.


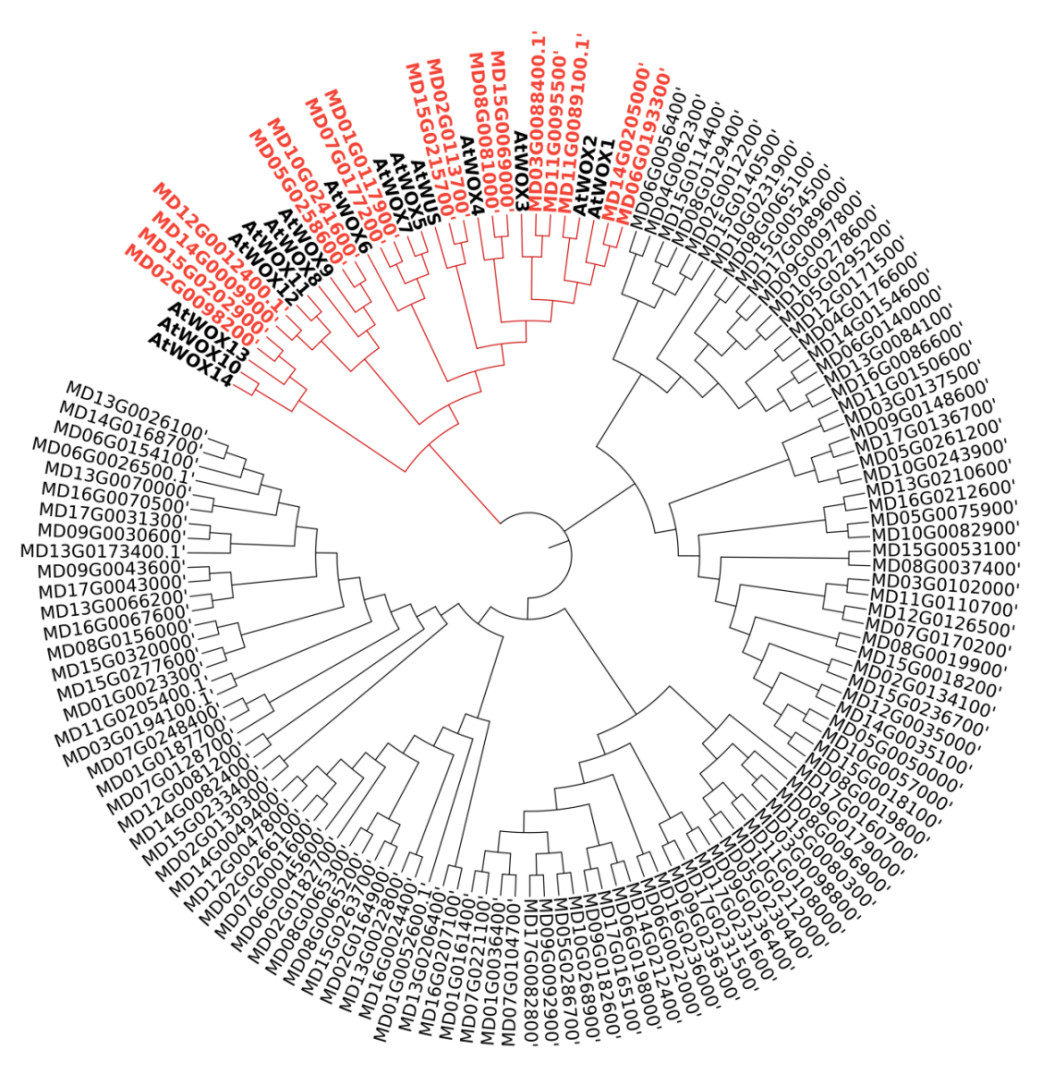


Fig. S2 The phylogenetic tree including apple MdHOX proteins and *Arabidopsis* WOX proteins*.* 17 *MdHOX* genes were marked in red and 15 *AtWOX* genes were in the same branch.


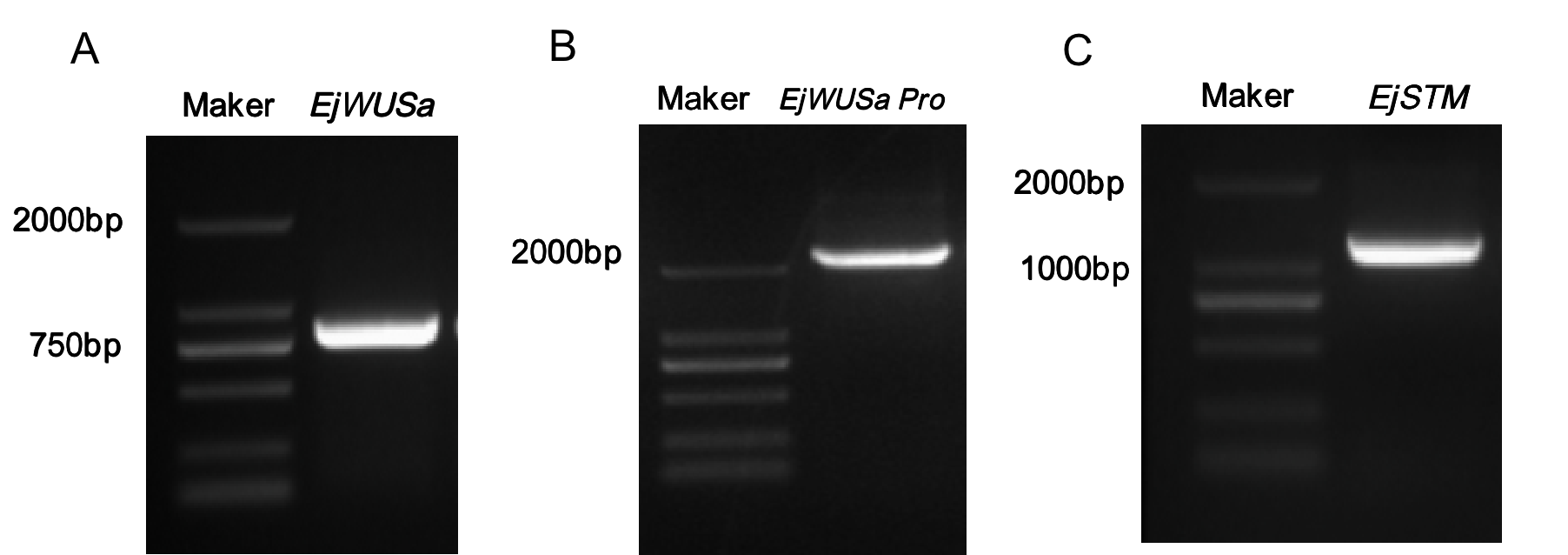


Fig. S3 The cloning of *EjWUSa*, *EjWUSa* promoter, and *EjSTM*. A: The cloning of *EjWUSa.* The open reading frame of *EjWUSa* is 987 bp. B: The cloning of the *EjWUSa* promoter*.* C*:* The cloning of *EjSTM.* The open reading frame of *EjSTM* is 1173 bp*.*


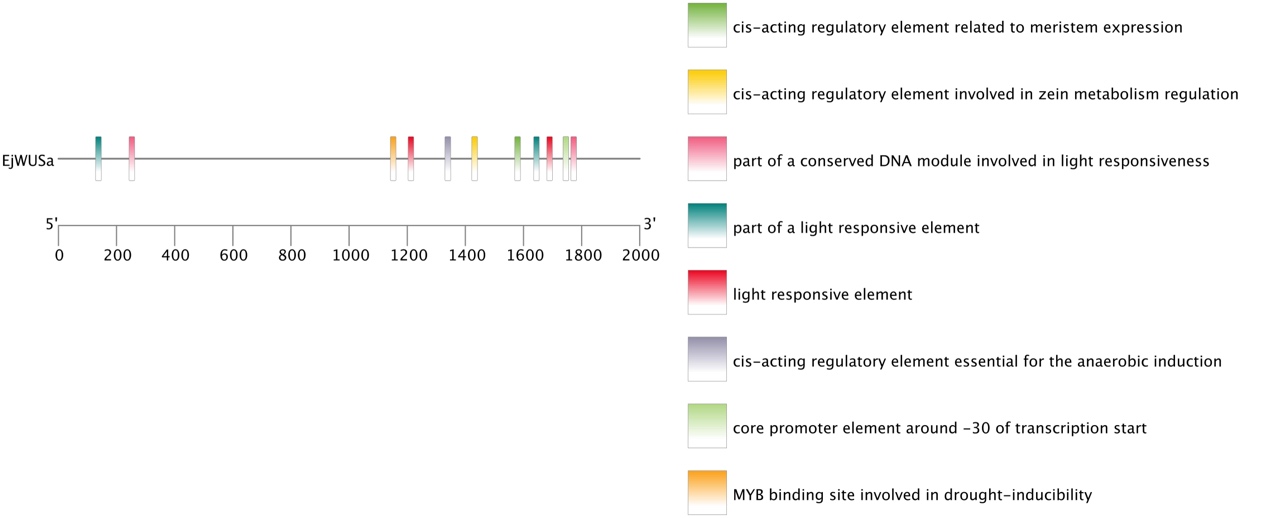


Fig. S4 The cis‐acting elements in the promoter of *EjWUSa*. Black lines indicate length. Colored squares indicate the binding elements on the *EjWUSa* promoter.


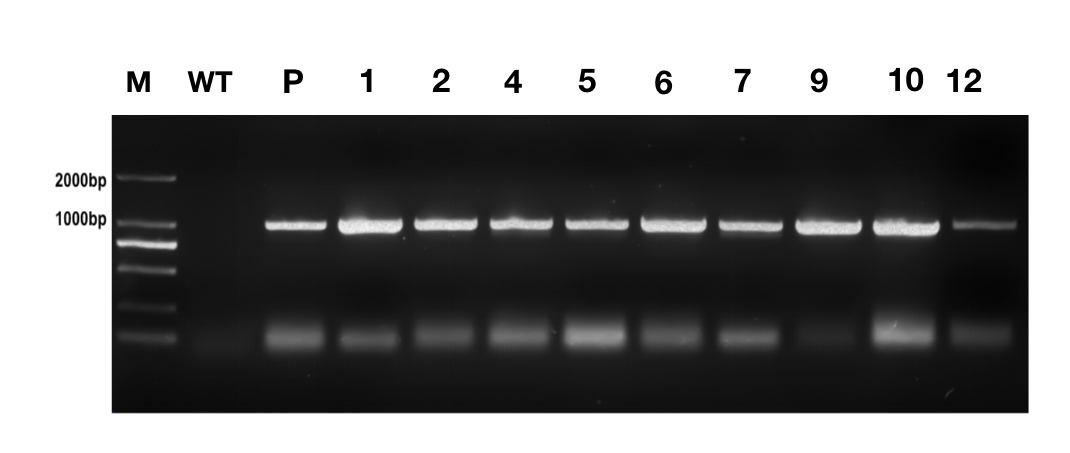


Fig. S5 The PCR detection of *EjWUSa* transgenic *Arabidopsis* and wild-type *Arabidopsis*. M: maker; WT: wild-type *Arabidopsis;* P*:* positive control.

Fig. S6 Multiple sequence alignment of the WOX proteins in apple. The identical amino acids are in black boxes.


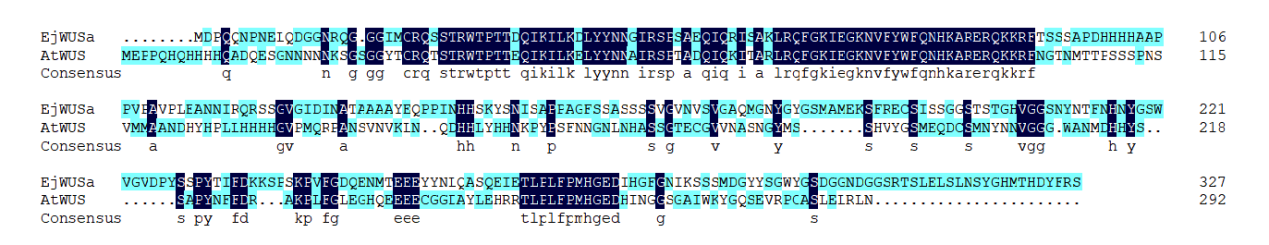
 Fig. S7 The sequence alignment of loquat EjWUSa protein and *Arabidopsis* AtWUS protein. The identical amino acids are in black boxes.
